# Tracking Decitabine Incorporation into Malignant Myeloid Cell DNA *in vitro* and *in vivo* by LC-MS/MS with Enzymatic Digestion

**DOI:** 10.1038/s41598-019-41070-y

**Published:** 2019-03-14

**Authors:** Sujatha Chilakala, Ye Feng, Lan Li, Reda Mahfouz, Ebrahem Quteba, Yogen Saunthararajah, Yan Xu

**Affiliations:** 1 Department of Chemistry, Cleveland State University, 2121 Euclid Avenue, Cleveland, Ohio 44115 USA; 2 Department of Hematology and Oncology, Taussig Cancer Institute, Cleveland Clinic, 2010 East 90th Street, Cleveland, OH 44195 USA

## Abstract

The DNA hypomethylating agents decitabine and 5-azacytidine are the only two drugs approved for treatment of all subtypes of the myeloid malignancy myelodysplastic syndromes (MDS). The key to drug activity is incorporation into target cell DNA, however, a practical method to measure this incorporation is un-available. Here, we report a sensitive and specific LC-MS/MS method to simultaneously measure decitabine incorporation and DNA hypomethylation. A stable heavy isotope of 2′-deoxycytidine was used as an internal standard and one-step multi-enzyme digestion was used to release the DNA bound drug. Enzyme-released decitabine along with other mononucleosides were separated by a reverse-phase C_18_ column and quantified by mass spectrometry using multiple-reaction-monitoring (MRM) mode, with a lower limit of quantitation at 1.00 nM. *In vitro* studies demonstrated dosage and time-dependent incorporation of decitabine into myeloid leukemia cell DNA that correlated with extent of DNA hypomethylation. When applied to clinical samples serially collected from MDS patients treated with decitabine, the method again demonstrated correlation between decitabine DNA-incorporation and DNA hypomethylation. This novel assay to measure the intended molecular pharmacodynamic effect of decitabine therapy can therefore potentially provide insights into mechanisms underlying sensitivity versus resistance to therapy.

## Introduction

Decitabine (5-aza-2′-deoxycytidine) is a nucleoside analog of 2′-deoxycytidine. Decitabine was introduced clinically four decades ago and was approved for the treatment of patients with myelodysplastic syndrome (MDS) in 2006 in the USA^1–4^. The only other drug approved to treat all subtypes of MDS is 5-azacytidine which is reduced in cells to the same active molecule, decitabine triphosphate, as decitabine. After parenteral administration, decitabine undergoes a three-step phosphorylation within cells into its active metabolite, decitabine triphosphate [first, to its monophosphate by deoxycytidine kinase (DCK); then to its diphosphate by deoxycytidine monophosphokinase; and finally to its triphosphate by nucleoside diphosphokinase] which is then directly incorporated by DNA polymerases into DNA during the S-phase of replication^5^. Decitabine triphosphate is the primary intracellular metabolite that has the antileukemic effect both *in vivo* and *in vitro*. Decitabine and its mono-phosphate can be rapidly metabolized by cytidine deaminase (CDA) and deoxycytidine deaminase (DCTD) respectively into uridine derivatives that do not have an epigenetic therapeutic effect^6^. After incorporation into DNA, decitabine inhibits DNA methylation by forming a covalent bond with DNA methyltransferase 1 (DNMT1). DNMT1 is a corepressor which generates an epigenetic repression (‘off’) mark by the addition of a methyl group to the fifth carbon of 2-deoxycytosine within the 5′-cytosine-guanosine (CpG) dinucleotides of DNA. This epigenetic mark generated by DNMT1 is robustly linked with gene repression and aberrant repression of tumor suppressor genes in malignant transformation^7–9^. Thus DNMT1-depletion by the DNA incorporated decitabine can reactivate tumor suppressor genes, leading to terminal differentiation or apoptosis of malignant cells^5,10^.

Target engagement is crucial for therapeutic efficacy of any drug. Since the incorporation of decitabine into DNA is essential for the epigenetic therapeutic effect, we postulate that quantitation of decitabine incorporation into cellular DNA can potentially guide individualization of therapy towards achievement of intended molecular pharmacodynamic effects, and provide insights into sensitivity versus resistance of malignant cells, which could be from failure to produce these intended molecular pharmacodynamic effects. Although decitabine incorporation into DNA has previously been measured using radioisotopic and liquid scintillation counting assays^11,12^, these previous methods had either limited sensitivity and used radioactive isotope labeled decitabine, which is not practical for use in clinical settings. LC-MS/MS method have been developed to quantify decitabine concentration in plasma^13^ however, this method is not applicable to measure decitabine incorporation in DNA and is less sensitive to use in clinical application. Another LC-MS/MS method has been developed to quantify total intracellular decitabine nucleotide concentrations, as well as DNA incorporation levels of decitabine^14^, but is less sensitive and correlation of DNA incorporation to hypomethylation was not demonstrated.

To address this unmet need, here we developed a sensitive LC-MS/MS method to simultaneously quantify incorporation of decitabine into DNA and DNA methylation, by measuring dG, dC, and 5mdC. The method was applied both *in vitro* and *in vivo*. The results demonstrated that this method can quantify the incorporation of decitabine into DNA, enabling quantification of the variation in the incorporation and response to treatment in different models and *in vivo*. This assay could be a useful tool for the purpose of understanding treatment sensitivity versus resistance and provide important guidance towards an overall goal of individualizing and optimizing therapy with this exclusive class of agent.

## Results

### Enzymatic hydrolysis of DNA

**Figure 1:**
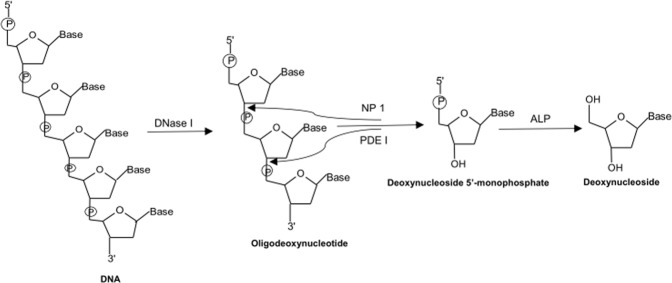
Schematic representation of DNA hydrolysis by a one-step tetra-enzyme digestion system that includes DNase 1, NP 1, PDE 1, and ALP.

In this work, the DNA samples were hydrolyzed by a one-step tetra-enzyme reaction (Fig. 1). This one-step enzyme reaction had been optimized and validated previously to retain 100% digestion efficiency in comparison to an optimized stepwise enzyme reaction^15^. Among the four enzymes employed, DNase I is an endonuclease that splits phosphodiester bonds of DNA and yields oligonucleotides with a free 3′-end hydroxyl group and a free 5′-end phosphate group; PDE I is a 3′ to 5′ exonuclease that successively hydrolyzes an oligonucleotide from 5′-end to 3′-end and produce deoxynucleoside 5′-phosphate; NP1 is a 5′ to 3′ exonuclease that acts in opposite direction to PDE I, and completely hydrolyzes an oligonucleotide from 3′-end to 5′-end to produce deoxynucleoside 5′-phosphate; and ALP is a hydrolase that hydrolyzes phosphate groups of deoxynucleotides to deoxynucleosides. Since the electrospray ionization tandem mass spectrometer (ESI-MS/MS) used for this work had much lower limits of quantitation for deoxynucleosides than those of deoxynucleotides; therefore, deoxynucleosides were the sought-after products for the enzyme digestion.

Enzyme digestion efficiency was evaluated by comparing the DNA concentration calculated from the amount of dG produced from DNA hydrolysis with the DNA concentration measured using UV spectroscopy. The rationale for the study is that DNA concentrations can be either measured by UV spectroscopy or calculated from the amount of a nucleoside released from hydrolysis and measured by LC-MS/MS^15^. In this work, the concentration of dG released from hydrolysis of calf DNA (250 mg/L measured by UV spectroscopy) of was used to calculate the concentration of DNA using eq. 1 (see Materials and Methods section) to evaluate the enzyme digestion efficiency. The DNA concentration (257 ± 4 mg/L, n = 5) calculated from the quantity of dG was similar to that measured by UV (250 ± 6 mg/L, n = 5), indicating that the enzymatic hydrolysis procedure used can completely hydrolyze DNA into individual nucleosides.

### Measurement of decitabine, dG, dC and 5mdC by LC-MS/MS

**Figure 2:**
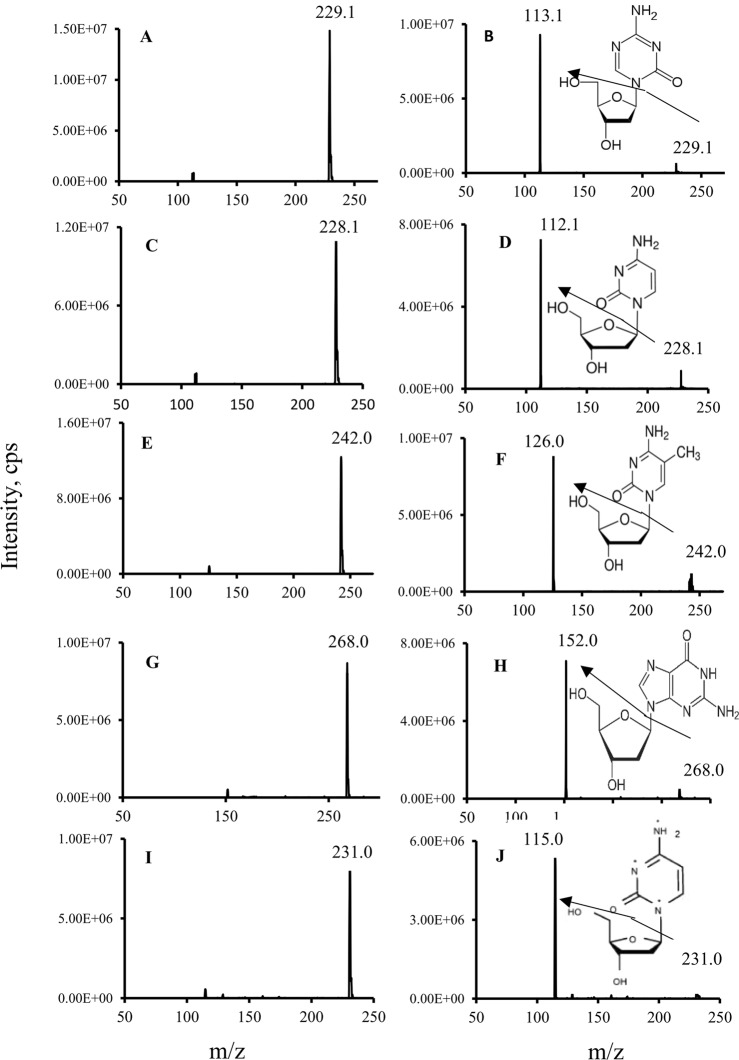
Representative mass spectra of precursor and product ions of decitabine, dC, 5mdC, dG and IS. (**A**) the precursor ion of decitabine; (**B**) the major product ion of decitabine; (**C**) the precursor ion of dC; (**D**) the major product ion of dC; (**E**) the precursor ion of 5mdC; (**F**) the major product ion of 5mdC; (**G**) the precursor ion of dG; (**H**) the major product ion of dG; (**H**) the precursor ion of IS; (**I**) the major product ion of IS.

The mass spectrometer was tuned by infusion of a mixture of decitabine, dC, 5mdC, dG and IS at each concentration of 500 ng/mL in the mobile phase to optimize both compound-dependent and source-dependent parameters. Since these deoxynucleoside were more easily to form protonated ions than deprotonated species in the acidic mobile phase, the positive ESI was used for the analytes’ identification and quantification. As shown in Fig. 2A,C,E,G,I, the predominated precursor ions of decitabine, dC, 5mdC, dG and IS were at m/z 229.1, 228.1, 242.0, 268.0 and 231.0, respectively. These precursor ions were broken into product ions by collision with nitrogen gas and produced the predominant product ions at m/z 113.1, 112.1, 126.0, 152.0 and 115.0, respectively (Fig. 2B,D,F,H,J). Therefore, the mass transitions of m/z 229.1 > 113.1, 228.1 > 112.1, 242.0 > 126.0, 268.0 > 152.0 and 231.0 > 115.0 for were chosen for the selective quantitation of decitabine, dC, 5mdC, dG and IS by MRM mode.

**Figure 3:**
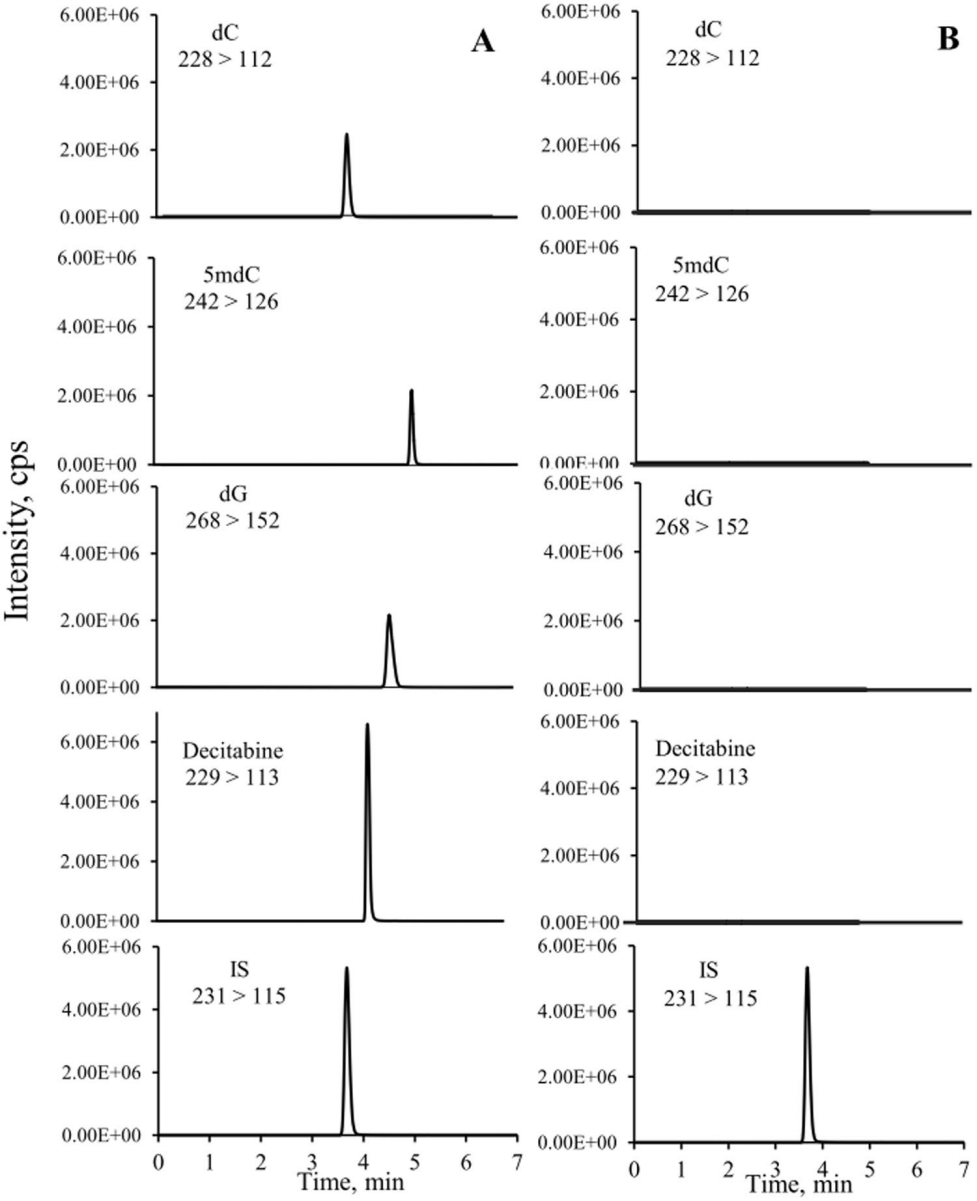
Representative MRM chromatograms of decitabine, dC, 5mdC, dG, and IS. (**A**) decitabine-treated DNA sample incubated with tetra enzymes; (**B**) decitabine-treated DNA sample incubated with buffer without tetra enzymes.

As shown in Fig. 3A, decitabine, dC, 5mdC and dG released by the enzyme digestion along with IS were separated and detected by our LC-MS/MS method in less than 6 min. The control studies showed that there were no contaminations observed from free nucleosides or unincorporated decitabine when DNA was incubated with digestion buffer without enzymes (Fig. 3B).

**Figure 4:**
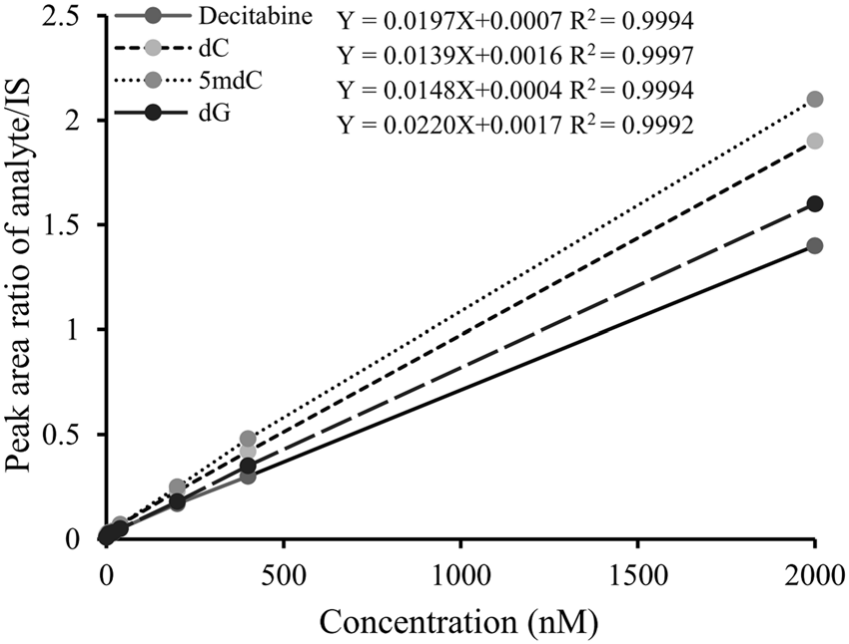
Calibration plots of decitabine, dC, 5mdC, and dG.

This method has a lower limit of quantification (LLOQ) of 1.00 nM for each analyte, which was defined by the lowest calibrator of a calibration plot; and a linear calibration range from LLOQ to 2.00 × 10^3^ nM (Fig. 4). Validation of the method was done according to the US-FDA guidance for industry on bioanalytical method validation^16^. The inter-day and intra-day accuracy and precision of the three mixed QC concentrations were ≤±6% and ≤8%, respectively (Table 1). The accuracy and precision of mixed calibrators over six calibration plots were ≤±6% and ≤11%, respectively (Table 2). These data were well within the acceptance ranges of US-FDA.

**Table 1:** Accuracy and precision of decitabine, dC, 5mdC and dG calibrators over six validation batches in digestion matrix.

Nominal [Analyte] (nM)	[Decitabine] (nM)			[dC] (nM)			[5mdC] (nM)			[dG] (nM)		
	[Measured ± SD^a^]	%RE^b^	CV^c^	[Measured ± SD^a^]	%RE^b^	CV^c^	[Measured ± SD^a^]	RE^b^	CV^c^	[Measured ± SD^a^]	%RE^b^	CV^c^
1.00	0.98 ± 0.09	−2	11	0.97 ± 0.07	−3	7	1.02 ± 0.06	2	6	0.97 ± 0.07	−3	7
4.00	4.1 ± 0.2	3	4	4.1 ± 0.2	2	4	3.9 ± 0.2	3	5	4.1 ± 0.2	3	5
20.0	20.7 ± 0.2	4	1	19.8 ± 0.5	−1	3	22 ± 1	10	5	19.6 ± 0.2	−2	1
40.0	40 ± 2	1	4	41 ± 3	3	7	42 ± 3	6	6	39 ± 1	−3	2
200	205 ± 4	3	2	196 ± 2	−2	1	194 ± 3	−3	8	203 ± 8	2	4
400	386 ± 1	−4	3	377 ± 2	–6	5	407 ± 1	2	2	412 ± 1	3	3
2.00 × 10^3^	(1.97 ± 0.02) × 10^3^	−1	1	(1.98 ± 0.02) × 10^3^	−1	1	(1.99 ± 0.02) × 10^3^	−0.4	3	(2.02 ± 0.02) × 10^3^	1	1

^a^Each measured value was calculated based on six measurements from different days.

^b^%RE = {(measured [analyte] − nominal [analyte])/nominal [analyte]} × 100%.

^c^CV = (standard deviation/mean value) × 100%.

**Table 2:** Intra- and inter-assay precision and accuracy.

Analyte	Nominal [QC] (nM)	Intra-assay^a^			Inter-assay^b^		
		[Measured QC ± SD] (nM)	%RE	CV	[Measured QC ± SD] (nM)	%RE	CV
Decitabine	3.00 (LQC)	3.2 ± 0.1	5	3	3.2 ± 0.1	6	4
	70.0 (MQC)	71 ± 2	3	2	71 ± 1	3	2
	1.60 × 10^3^ (HQC)	(1.57 ± 0.02) × 10^3^	−2	1	1.56 ± 0.01) × 10^3^	−2	2
dC	3.00 (LQC)	2.9 ± 0.1	−2	4	2.9 ± 0.2	−1	6
	70.0 (MQC)	69 ± 3	2	4	72 ± 2	4	3
	1.60 × 10^3^ (HQC)	(1.63 ± 0.02) × 10^3^	2	1	(1.65 ± 0.02) × 10^3^	3	2
5mdC	3.00 (LQC)	3.1 ± 0.1	5	4	3.2 ± 0.3	6	8
	70.0 (MQC)	71 ± 4	2	6	72 ± 3	3	4
	1.60 × 10^3^ (HQC)	(1.58 ± 0.02) × 10^3^	−1	1	(1.58 ± 0.02) × 10^3^	−1	1
dG	3.00 (LQC)	3.2 ± 0.1	6	4	3.1 ± 0.2	3	6
	70.0 (MQC)	66 ± 3	−5	4	67 ± 2	−3	3
	1.60 × 10^3^ (HQC)	(1.61 ± 0.02) × 10^3^	1	1	(1.61 ± 0.02) × 10^3^	1	2

^a^Intra-assay precision and accuracy were assessed by five replicate measurements of individual QC at each concentration.

^b^Inter-assay precision and accuracy were assessed by five parallel measurements of five identical QCs at each concentration.

The studies of matrix effect and analyte recovery on all analytes were conducted using mixed QC at three concentrations (Table 3). For all analytes, the absolute matrix factors ranged 0.933–1.03 and the IS normalized matrix factors ranged 0.955–1.03; and the absolute recoveries ranged 90.3–100% and the IS normalized recoveries ranged 96–102%. These data indicated there was neither significant matrix effect from enzyme digestion buffer nor significant difference in recoveries of analytes between enzyme digestion buffer and the mobile phase of LC separation, and the preparation of mixed calibrators of decitabine, dC, dG and 5mdC in either enzyme digestion buffer or the LC mobile phase makes no difference in the analytical results. Therefore, the mixed calibrators of dC, dG and 5mdC prepared in the mobile phase were adopted for the method.

**Table 3:** Matrix effect and recovery of decitabine from enzyme digestion matrix (n = 5).

[Decitabine] (nM)				[dC] (nM)			[5mdC] (nM)			[dG] (nM)		
	3.00 (LQC)	70.0 (MQC)	1.60 × 10^3^ (HQC)	3.00 (LQC)	70.0 (MQC)	1.60 × 10^3^ (HQC)	3.00 (LQC)	70.0 (MQC)	1.60 × 10^3^ (HQC)	3.00 (LQC)	70.0 (MQC)	1.60 × 10^3^ (HQC)
MF_Ana_^a^ ± SD	0.959 ± 0.001	0.972 ± 0.001	1.01 ± 0.00	0.964 ± 0.002	0.976 ± 0.002	1.01 ± 0.03	0.933 ± 0.003	0.951 ± 0.006	0.968 ± 0.003	0.943 ± 0.003	0.961 ± 0.007	0.990 ± 0.002
MF_IS_^b^ ± SD	0.977 ± 0.002	0.981 ± 0.001	0.98 ± 0.01	0.977 ± 0.002	0.981 ± 0.001	0.98 ± 0.01	0.977 ± 0.002	0.981 ± 0.001	0.98 ± 0.01	0.977 ± 0.002	0.981 ± 0.001	0.98 ± 0.01
IS Normalized MF^c^ ± SD	0.982 ± 0.002	0.991 ± 0.001	1.03 ± 0.01	0.987 ± 0.003	0.995 ± 0.002	1.03 ± 0.03	0.955 ± 0.004	0.970 ± 0.006	0.988 ± 0.01	0.965 ± 0.004	0.980 ± 0.007	1.01 ± 0.01
R_Ana_^d^ ± SD (%)	91.2 ± 0.2	97 ± 1	100 ± 1	92.6 ± 0.3	98 ± 2	100 ± 2	90.3 ± 0.4	95. ± 3	98 ± 1	90.7 ± 0.5	96 ± 4	99 ± 1
R_IS_^e^ ± SD (%)	91 ± 2	99 ± 3	99 ± 2	91 ± 2	99 ± 3	99 ± 2	91 ± 2	99 ± 3	99 ± 2	91 ± 2	99 ± 3	99 ± 2
IS Normalized R^f^ ± SD	1.00 ± 0.02	0.98 ± 0.03	1.01 ± 0.02	1.02 ± 0.0	0.99 ± 0.04	1.01 ± 0.03	0.99 ± 0.02	0.96 ± 0.04	0.99 ± 0.02	1.00 ± 0.02	0.97 ± 0.05	1.00 ± 0.02

^a^MF_Ana_ = (mean peak area of analyte in extracted enzyme digestion matrix)/(mean peak area of analyte in the mobile phase).

^b^MF_IS_ = (mean peak area of IS in extracted enzyme digestion matrix)/(mean peak area of IS in the mobile phase).

^c^IS Normalized MF = MF_Ana_/MF_IS_.

^d^R_Ana_ = [(mean peak area of analyte in enzyme digestion matrix)/(mean peak area of analyte in extracted enzyme digestion matrix)] × 100%.

^e^R_IS_ = [(mean peak area of IS in enzyme digestion matrix)/(mean peak area of IS in extracted enzyme digestion matrix)] × 100%.

^f^IS Normalized R = (R_Ana_/R_IS_) × 100%.

Note: IS 2′-Deoxycytidine ^15^N_3_ added at a concentration of 200 nM.

### Decitabine incorporation vs. hypomethylation of DNA in leukemia cell lines

The LC-MS/MS method developed was first tested by human leukemia cell lines HL-60 and U937 for quantitation of decitabine incorporation in cellular DNA and cell responsiveness to decitabine treatment (*i*.*e*., decrease of DNA methylation or DNA hypomethylation). In this study, the effects of drug dose (0.000, 0.100, 0.500, 1.00 and 10.0 μM decitabine) and treatment times (24- and 48-h incubation) were investigated. The cellular DNAs were isolated from samples of each dose and time point. The enzyme-released decitabine, dC, dG and 5mdC from the cellular DNA were determined by the method developed and percent methylations were calculated.

**Figure 5:**
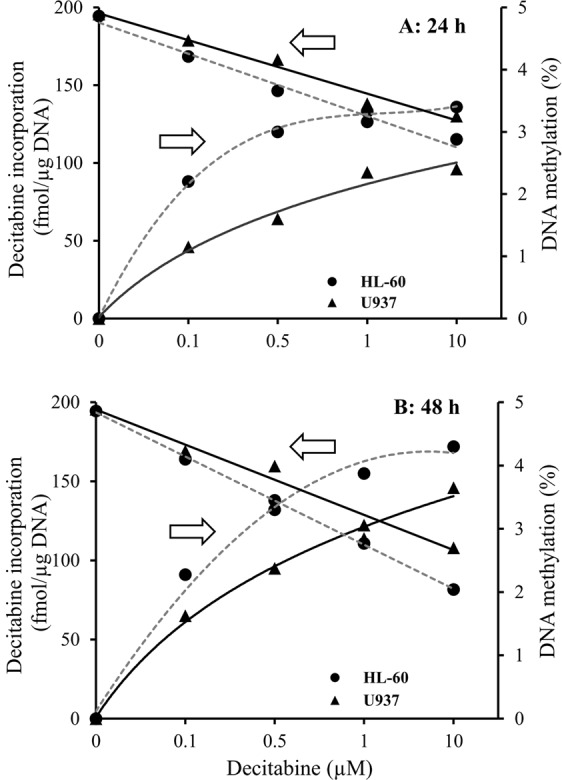
Decitabine incorporation correlates with DNA demethylation in HL-60 and U-937 cells. (**A**) 24-h treatment, and (**B**) 48-h treatment. Experimental conditions were described in “*In vitro* study” under the section of “Materials and Methods”.

As shown in Fig. 5 and summarized in Table 4, the amounts of decitabine incorporated in HL-60 and U937 DNAs were directly proportional to the drug dose and the treatment time. The higher dose and the longer treatment time produced larger amounts of decitabine incorporation in DNA and greater degree of DNA hypomethylation. Also, HL-60 cells have greater viability than U937 cells. This was revealed by the DNA extracted from each of the cell lines after 24-h and 48-h treatment with decitabine. On average, the amounts of DNA produced by HL-60 cells were 1.3 and 2.0 times higher than those of U937 cells after 24-h and 48-h treatment with decitabine. Therefore, HL-60 cells showed more sensitivity to the drug treatment in comparison to U937 with larger decitabine incorporation and greater degree of DNA hypomehtylation, which suggested that for U937 cell line, it may need higher decitabine exposure (either dose or time) than HL-60 cell line to reach the same drug effect.

**Table 4:** The effects of decitabine dosage and treatment time on decitabine incorporation in DNA and DNA hypomethylation.

Decitabine dose (µM)	24-h Study				48-h Study			
	Decitabine incorporation (fmol/mg DNA)		DNA methylation		Decitabine incorporation (fmol/mg DNA)		DNA methylation	
	HL-60	U937	HL-60	U937	HL-60	U937	HL-60	U937
0.000	0.000	0.000	4.86	4.89	0.000	0.000	4.86	4.89
0.100	88.0	46.0	4.21	4.47	96.0	65.0	4.10	4.23
0.500	120	64.0	3.66	4.16	132	95.0	3.45	3.99
1.00	133	94.0	3.16	3.45	155	114	2.77	3.06
10.0	136	96.0	2.88	3.25	172	146	2.04	2.70

**Figure 6:**
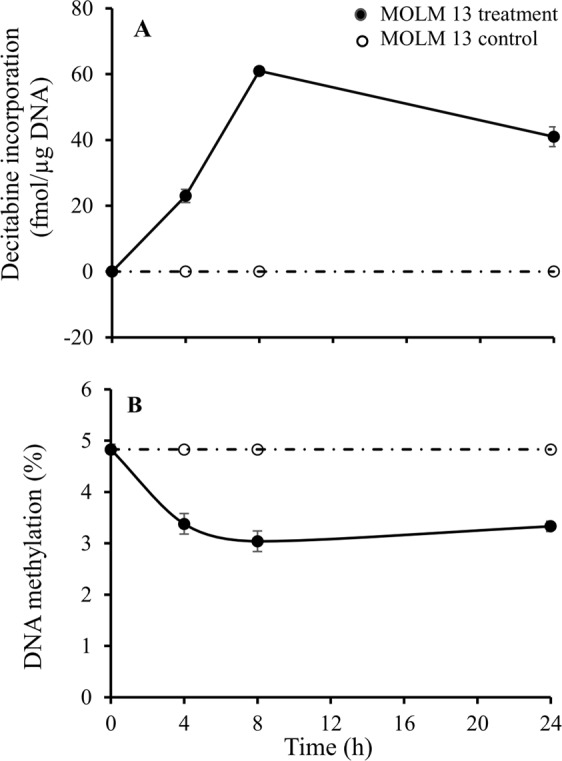
Time course study of decitabine incorporation and DNA demethylation in MOLM-13 cells. (**A**) Decitabine incorporation profiles, and (**B**) DNA demethylation profiles. Experimental conditions were described in “*In vitro* study” under the section of “Materials and Methods”.

Decitabine incorporation and hypomethylation effect in a different human leukemia cell line MOLM-13 were also investigated using both a single dose of decitabine (0.500 μM) for the treatment group and an equal volume of vehicle (PBS 1X, pH 7.4) for the control group at various time points (0, 4, 8, and 24 h). As shown in Fig. 6, the profiles of drug effect in the cell line could be monitored by the time course, and the maximum amount of drug incorporation and hypomethylation effect were observed at 8 h in MOLM-13 cells.

### Decitabine incorporation vs. hypomethylation of DNA in animal study

**Figure 7:**
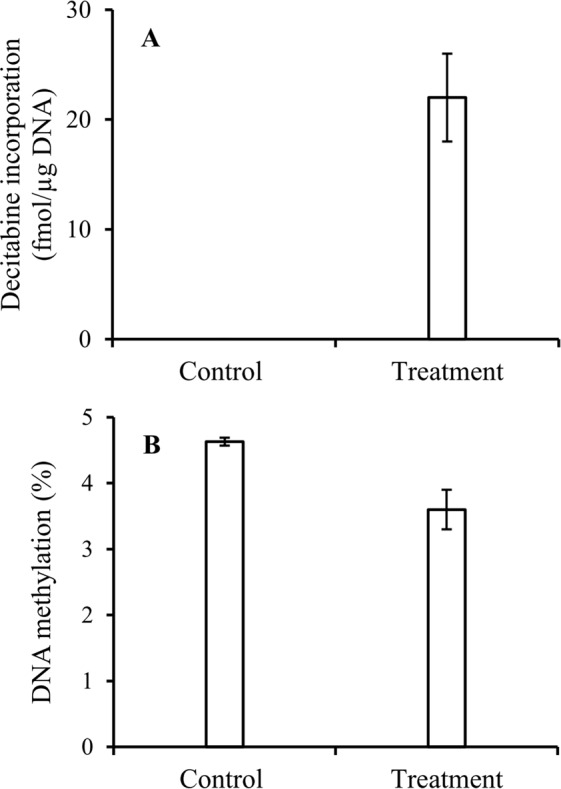
Decitabine incorporation and DNA demethylation *in vivo* animal study. (**A**) Decitabine incorporation in the controls and decitabine-treated mice (n = 4), and (**B**) DNA demethylation in the controls and decitabine-treated mice. Experimental conditions were described in “*In vivo* animal study” under the section of “Materials and Methods”.

The method developed was further tested by analysis of DNA extracted from bone marrow of NSG mice inoculated with human primary AML cells for quantitative measurement of DNA-incorporated decitabine and DNA hypomethylation. The experimental details were described in “*In vivo* animal study” section under “Materials and Methods”. As shown in Fig. 7A, decitabine incorporation in bone marrow cells from four genetically identical mice after the drug treatment ranged 18.4 to 25.9 fmol per µg DNA, and such treatment significantly reduced DNA methylation in all mice (*P* = 0.006, *t*-test) (Fig. 7B). We had previously reported the efficacy of this treatment in reducing leukemia burden^17,18^.

### Decitabine incorporation vs. hypomethylation of DNA in clinical study

**Figure 8:**
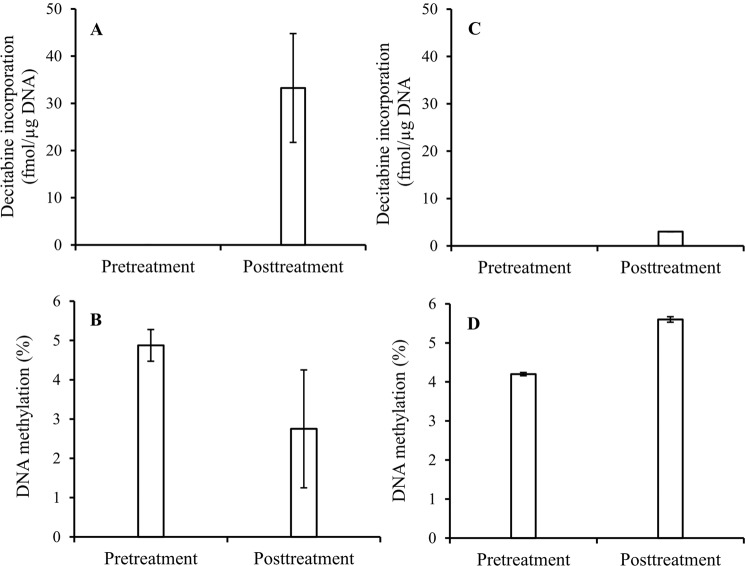
Decitabine incorporation and DNA demethylation in clinical study. (**A**) Decitabine incorporation in AML patients who were sensitive to decitabine treatment (n = 4), (**B**) DNA demethylation in AML patients who responded to decitabine treatment (n = 4) (*P* = 0.07, *t*-test), (**C**) Decitabine incorporation in AML patient who did not respond to decitabine treatment, and D) DNA demethylation in AML patient who did not respond to decitabine treatment. Experimental conditions were described in “Clinical study” under the section of “Materials and Methods”.

Finally, the method developed was applied to PBMCs isolated from peripheral blood obtained from five MDS patients before and after six weeks of decitabine treatment by a very low dose (0.2 mg/kg) subcutaneous regimen administered twice per week^2^. The experimental details were described in “Clinical study” section under “Materials and Methods”. Among the five patients, four responded well to the decitabine therapy, and one did not. The molecular mechanism of decitabine action and drug resistance in these patients would be depicted by Fig. 8. For patients #1 to #4 who were sensitive to decitabine treatment, decitabine incorporation and DNA hypomethylation were large and significant (*P* = 0.07, *t*-test) (Fig. 8A,B); whereas for patient #5 who was resistant to the drug, there were minimum decitabine incorporation (Fig. 8C), and even higher DNA methylation in posttreatment (Fig. 8D).

## Discussion

Target engagement is necessary for any drug to work, and the measurement of target engagement of decitabine (*i*.*e*., decitabine incorporation and DNA hypomethylation) is the first step to understand patients’ sensitivity and resistance in decitabine therapy; however, a practical method for this purpose was lacking. The method we developed is practical, very sensitive and specific for measurement of decitabine, 5mdC, and dG, as no interferences from endogenous molecules were observed. Furthermore, this method has been fully validated in enzyme digestion matrix and essentially met the validation criteria established by US-FDA. Such method may help us not only to understand drug action and resistance mechanisms of decitabine, but also to design more effective dosing regimens for patients.

In the *in vitro* dose and time course studies, no cell death was observed with the treatment doses and durations used. Decitabine incorporation into DNA of HL-60 and U937 was both dose and time dependent, and notably, uptake via passive nucleoside transporters 1 (ENT1)^19^ is drug concentration-dependent. Although decitabine triphosphate incorporation into DNA by DNA polymerase occurs only during S-phase^20^, the drug incorporation in the *in vitro* studies was around-the-clock because cycling by the cells was unsynchronized. In the time course study of decitabine incorporation in DNA of MOLM-13 cells (Fig. 6), the decitabine incorporation profile deviated from the steady-state profile, likely because of decomposition of decitabine in cell culture medium (decitabine has an *in vitro* half-life of 5–16 hours at 37 °C)^21^.

Different responses to decitabine treatments in *in vitro* and *in vivo* may be a consequence of differences in expression of key pyrimidine metabolism enzymes in different tissues and different models^17,18^. DCK performs the initial phosphorylation of decitabine and rate-limits the conversion of decitabine to decitabine triphosphate. CDA and DCTD metabolize decitabine and, decitabine mono-phosphates into non-DNMT1-depleting uridine derivatives. In the case of the patient with the myeloid malignancy that did not respond to decitabine, decitabine triphosphate incorporation into DNA was about 9% (3 fmol/μg DNA) in comparison to ~10-fold greater incorporation in the patients with disease that did respond to treatment (33 ± 11 fmol/μg DNA); furthermore, in the patient with unresponsive disease, DNA hypomethylation was not observed. Instead DNA methylation increased from 4.22% (pretreatment) to 5.60% (posttreatment)^3,8^.

Previous attempts at correlating DNA hypomethylation in peripheral blood cells with response to decitabine treatment have had mixed results^22–24^ One difference is that these previous studies administered decitabine by standard pulse-cycled regimens, and DNA methylation changes may therefore have been more substantially influenced by the timing between peripheral blood collection and drug administration. By contrast, the patients analyzed in this study received decitabine twice weekly continuously for 6 weeks prior to the peripheral blood sample collection. A limitation of our study and on our conclusions remains the small number of clinical samples analyzed.

In sum, quantitative measurement of decitabine incorporation rates and DNA hypomethylation effects can potentially determine early time points where patients with disease are non-responsive to their decitabine dosing regimens, and guide dosage adjustment or selection of alternative therapies. In other words, since the molecular pharmacodynamic intention with decitabine therapy is its incorporation into DNA, it may be useful to prospectively evaluate if the method for measurement of this pharmacodynamic effect that we describe here can guide individualization and optimization of therapy in patients with myeloid malignancies.

## Materials and Methods

### Chemicals

Decitabine was obtained from Developmental Therapeutics Program of the National Cancer Institute (Bethesda, MD, USA). Bis(2-hydroxyethyl)amino-tris(hydroxymethyl)methane (BIS-TRIS), deoxyribonuclease I type II (DNase I), nuclease P1 (NP1), bovine alkaline phosphatase (ALP), 2′-deoxycytidine (dC), 2′-deoxyguanosine (dG), 2′-deoxythymidime (dT), 2′-deoxyadenosine (dA) and formic acid were obtained from Sigma-Aldrich (St. Louis, MO, USA). Snake venom phosphodiesterase I (PDE I) was obtained from Worthington Biochemical (Lakewood, NJ, USA). 2′-Deoxycytidine ^15^N_3_ (96–98%, a stable heavy isotope of 2′-deoxycytidine) was purchased from Synthèse AptoChem (Montreal, Quebec, Canada). 5-methyl-2′-deoxycytidine (5mdC) was purchased from Santa Cruz Biotechnology (Dallas, TX, USA). Sodium chloride, zinc chloride, phosphate buffered saline (PBS, 10X, pH 7.4), phenol saturated with Tris buffer (pH 6.6), HPLC-grade of methanol, acetonitrile and chloroform were purchased from Fisher Scientific (Fair Lawn, NJ, USA). 10% sodium dodecyl sulfate (SDS) solution was obtained from Bio-Rad Laboratories (Hercules, CA, USA). RPMI-1640 medium with L-glutamine was purchased from Mediatech (Manassas, VA, USA). Fetal bovine serum was purchased from GE Healthcare Life Sciences (Logan, UT, US). RiboShredder™ RNase Blend was purchased from Epicentre (Madison, WI, USA). Proteinase K was ordered from Qiagen (Valencia, CA, USA).

### *In vitro* studies

Cell lines HL-60 and U937 were obtained from American Type Culture Collection (Rockville, MD, USA). MOLM-13 cell line was obtained from Leibniz Institute DSMZ - German Collection of Microorganisms and Cell Cultures (Braunschweig, Germany). HL-60, U937 and MOLM-13 cell lines were cultured in RPMI-1640 medium supplemented with L-glutamine, 1% Penicillin-Streptomycin and 10% (v/v) fetal bovine serum in a humidified 5% CO_2_ incubator at 37 °C. Decitabine solution was freshly prepared for each experiment by dissolving decitabine powder in PBS (1X, pH 7.4) at a concentration of 1.00 mM.

For each 1 × 10^6^ cells of HL-60 and U937 cell lines, the dosages of decitabine added to the culture media are 0.00, 0.100, 0.500, 1.00, or 10.0 μM; and were treated for 24 h and 48 h at 37 °C in a humidified 5% CO_2_ incubator. MOLM-13 cells (1 × 10^6^ cells) was treated with 0.500 μM of decitabine or an equal volume of PBS (1X, pH 7.4) as control for 0, 4, 8, and 24 h at 37 °C in a humidified 5% CO_2_ incubator. After treatment, the cells were removed from the medium by centrifugation at 1,500 × g, 4 °C for 5 min; and washed twice with 5.00 mL of PBS (1X, pH 7.4) each. The cell pellets were collected and stored in −20 °C until DNA extraction.

### *In vivo* animal study

The animal study for this work was approved by the Cleveland Clinic Institutional Animal Care and Use Committees (IACUC) and all methods were performed in accordance with the relevant guidelines and regulations. NSG mice were obtained from Jackson Laboratory (Bar Harbor, ME, USA). Primary acute myelogenous leukemia (AML) cells from patients were transplanted by tail-vein injection (0.4 × 10^6^ cells/mouse) into non-irradiated 6–8 week old NSG mice (n = 4 per/group). Mice were anesthetized with isoflurane before transplantation. On day 15 of inoculation, animal groups were treated subcutaneously with vehicle (PBS 1X, pH 7.4) or decitabine 0.100 mg/kg for three consecutive days per week during the course of study. The control-group animals (median survival of 45 days) were euthanized by an IACUC approved method for signs of distress, and the treatment-group animals were continued with treatment for 90 days. At 90 day of the treatment, animals were euthanized by the IACUC approved method.

DNA was extracted from bone marrow using the procedure published in the ref.^25^. Bone marrow cells were washed twice with 10.0 mL PBS (1X, pH 7.4) each. In each wash step, the cells were gently vortexed in PBS (1X, pH 7.4) for 1 min and then centrifuged down at 1,500 × g, 4 °C, for 10 min. After washing, the cell pellet was frozen at −20 °C until DNA extraction.

### Clinical study

The five MDS patients of this study were enrolled in the clinical trial (NCT01165996) with written informed consent on a protocol approved by the Institutional Review Board of Taussig Cancer Institute at the Cleveland Clinic and all methods were performed in accordance with the relevant guidelines and regulations. The patients analyzed had MDS sub-types chronic myelomonocytic leukemia (CMML, n = 3) and refractory cytopenia with multi-lineage dysplasia (RCMD, n = 2). The decitabine regimen was 0.2 mg/kg administered subcutaneously two consecutive days per week with the post-treatment samples obtained after six weeks of this treatment. 10.0 mL of peripheral blood samples were collected from each patient in heparinized BD Vacutainer® tubes (Becton, Dickinson and Company, Franklin Lakes, NJ, USA) before and at the end of 6^th^ week of treatment. The blood samples were fractioned by the Ficoll-Paque method^26^. Briefly, 10.0 mL heparinized blood was layered on the top of 12.0 mL Ficoll-Paque Plus reagent (GE Healthcare Bio-Sciences, Piscataway, NJ, USA) in a 50.0-mL sterile polypropylene centrifuge tube (RNase/DNase free); then the tube was centrifuged at 300 × g, 4 °C, for 20 min. The peripheral blood mononuclear cells (PBMCs) at the interface between plasma and polymorphonuclear cells were collected and transferred into a clean 15.0-mL centrifuge tube and washed twice with 10.0 mL PBS (1X, pH 7.4) each. In each wash step, the PBMCs were gently vortexed in PBS (1X, pH 7.4) for 1 min and then centrifuged down at 4 °C and 1,500 × g for 10 min. After washing, the cell pellet was collected and stored in −20 °C until DNA extraction.

### DNA isolation and hydrolysis

Each cell pellet collected from the *in vitro*, *in vivo* and clinical studies was lysed by mixing gently with 2.00 mL of TE buffer (containing 10.0 mM Tris and 1.00 mM EDTA at pH 8.0) and 240 μL of 10% SDS solution for 2 min. Then, the lysate was incubated with 25.0 μL of proteinase K (600 mAU/mL) for 1 h at 37 °C. After the incubation, the sample solution was transferred to a phase lock gel tube (5 Prime, Gaithersburg, MD, USA), and DNA was extracted using standard phenol/chloroform extraction method^27^. The DNA extracted was dissolved in 5.00 mM BIS-TRIS buffer (pH 7.0) to a concentration of 1.00 mg/mL measured by UV spectrophotometry. The co-extracted RNA was removed by adding 2.00 µL of RiboShredder™ RNase Blend (1 U/µL) to each 100 µL of DNA sample (1.00 mg/mL). After a 30-min incubation at 37 °C, the sample was mixed with 1.00 mL of pre-chilled ethanol (−20 °C) and kept at −20 °C for overnight to precipitate the DNA in the sample. After centrifugation at 15,000 × g for 15 min, the supernatant was discarded, and the DNA pellet was washed with 1.00 mL of pre-cooled 70% ethanol (−20 °C) twice. Then, the DNA pellet was air-dried and reconstituted with 100 µL of 5.00 mM BIS-TRIS buffer (pH 7.0) for subsequent enzyme digestion.

DNA hydrolysis was performed by denaturing ds-DNA to ss-DNA in boiling water for 30 min followed by a one-step tetra-enzyme digestion^15,28^. The tetra-enzyme cocktail was prepared by mixing the enzyme solutions as follows: 10.0 μL of DNase I (20,000 U/mL), 15.0 μL of NP1 (200 U/mL), 40.0 μL of PDE I (100 U/mL) and 0.50 μL of ALP (40,000 U/mL). For each 50.0 μL of DNA (0.500 μg/μL for the measurement of DNA incorporated decitabine; 1.25 ng/μL for the measurements of dC, dG, and 5mdC), 4.00 μL of the tetra-enzyme cocktail was added and mixed well; then, the mixture was placed in a 37 °C water bath for overnight incubation to secure a complete digestion of DNA to mononucleosides. After incubation, 5.00 μL of 2′-deoxycytidine ^15^N_3_ (the internal standard, IS, 2.00 µM in the LC mobile phase) was added to a digested sample and vortex-mixed for 30 s. Each DNA digest was deproteinized by 450 μL of acetonitrile, then centrifuged at 15,000 x g for 10 min. 450 μL of supernatant was collected and evaporated to dryness at 30 °C for 60 min in a TurboVap® LV Evaporator (Zymark, Hopkinton, MA, USA) under a pressurized stream of nitrogen gas (20 psi). The dried residue was reconstituted in 50.0 μL of the LC mobile phase and subjected to LC-MS/MS analysis.

### LC-MS/MS system

The LC-MS/MS instrumentation used for this work consisted of a Prominence UFLC system (Shimadzu, Columbia, MD, USA) for analyte separation and a QTRAP 5500 tandem mass spectrometer (AB Sciex, Foster City, CA, USA) for quantitation. The system was controlled by AB Sciex Analyst® software (version 1.6.1) for its operation, data acquisition, and processing. The UFLC system included a system controller (CBM-20A), two binary pumps (LC-20AD), a temperature-controlled autosampler (SIL 20AHT) and an online degasser (DGU20A3).

### Quantitation of decitabine, dG, dC, and 5mdC

The LC separation was carried out on a Hypersil Gold aQ C18 column (50 × 2.1 mm, 3 µm) (Thermo Scientific, Waltham, MA, USA) with a Hypersil Gold aQ C18 guard column (10 × 2.1 mm, 3 μm) using the LC mobile phase consisting of 0.1% formic acid aqueous solution and methanol (87.5:12.5, v/v) at a flow rate of 0.300 mL/min. For each analysis, 10.0 μL of sample was injected into the system by autosampler set at 4 °C. The LC elute was introduced into the mass spectrometer operated under the positive turbo-ion spray ionization mode. The optimized instrument parameters were as follows: curtain (CUR), 40 psi; collision activated dissociation (CAD) gas, medium; nebulizer gas (GS1), 40 psi; turbo heater gas (GS2), 45 psi; turbo ion spray voltage (IS), +5200 V; source temperature (TEM), 300 °C; declustering potential (DP), 35 V; entrance potential (EP), 7 V; collision energy (CE), 15 V; collision cell exit potential (CXP), 18 V; mass resolutions (Q1 and Q3), 1 unit. The multiple-reaction-monitoring (MRM) mode was used for analyte quantitation.

Mixed calibrators of decitabine, dC, dG and 5mdC (1.00, 4.00, 20.0, 40.0, 200, 400 and 2.00 × 10^3^ nM) together with single- and double- blanks, and mixed QCs (3.00, 70.0 and 1.60 × 10^3^ nM) were prepared in 5.00 mM BIS-TRIS buffer (pH 7.0) and subjected to the same sample preparation procedure as DNA samples. The linear calibration plots for decitabine, dC, dG, and 5mdC were constructed using peak area ratios (y) of each analyte to the IS versus the concentrations of calibrators (x) with 1/x weighting, and the least squares linear regression equations were obtained as the calibration equations for individual analytes.

The concentrations of decitabine, dC, dG, and 5mdC in each unknown sample were back calculated by the calibration equations using the peak-area ratios of each analyte in the unknown sample to that of the IS. The accurate mass concentration of DNA was determined using the measured molar concentration of dG by the following equation:

1 $$[{\rm{DNA}}]\,({\rm{mg}}/{\rm{L}})=[{\rm{dG}}]\,\times \,{\rm{618}}\,({\rm{g}}/{\rm{mol}})/0.41$$

where [dG] is the measured concentration of dG in mM; 618 (g/mol) is the molar mass of G/C pair, and 0.41 is the percentage of G/C pair in human DNA^29^. The amount of DNA incorporated decitabine was expressed as pmol of decitabine per μg of DNA. The percent methylation was calculated using the following equation^29–31^:

2 $$ \% \,{\rm{methylation}}=[5{\rm{mdC}}/({\rm{dC}}+5{\rm{mdC}})]\times 100 \% $$

### Statistical analysis

Statistical analysis was performed using GraphPad Prism 5.0 (GraphPad Software Inc., San Diego, CA).

## Conclusion

We have developed a sensitive and specific LC-MS/MS method for quantitative determination of decitabine DNA incorporation and hypomethylation effect both *in vitro* and *in vivo*. This method is practical and can be easily adopted in preclinical and clinical studies of decitabine. The preliminary data from decitabine-treated AML patients show that this method not only allows us to quantitatively measure the pharmacokinetic endpoints of decitabine but also provides supporting evidence for drug action mechanisms on patients’ sensitivity and resistance in decitabine therapy. It may be useful for determining suitable decitabine dosing regimens for individual patients to achieve desirable therapy outcomes.
